# Supplementary material for: Probing the pinning strength of magnetic vortex cores with sub-nanometer resolution
Source: Nat Commun. 2020 Jun 5;11:2833. doi: 10.1038/s41467-020-16701-y (PMC7275073; doi:10.1038/s41467-020-16701-y)
Supplement: Supplementary file 1 — Supplementary Information [file 41467_2020_16701_MOESM1_ESM.pdf]

Supplementary Information: Probing the Pinning Strength of Magnetic Vortex Cores with sub-nm Resolution

Christian Holl,[1,*] Marvin Knol,[1] Marco Pratzer,[1] Jonathan Chico,[2]
Imara Lima Fernandes,[2] Samir Lounis,[2] and Markus Morgenstern[1]

[1]*II. Institute of Physics B and JARA-FIT,*
RWTH Aachen University, D-52074 Aachen, Germany
[2]*Peter Grünberg Institut and Institute for Advanced Simulation,*
Forschungszentrum Jülich and JARA, 52425, Jülich, Germany

(Dated: May 11, 2020)

* holl@physik.rwth-aachen.de

CONTENTS

Supplementary Note 1. Fe island 3

Supplementary Note 2. Micromagnetic energy densities of vortex core 4

Supplementary Note 3. Core fitting procedure 5

Supplementary Note 4. Core movement in elliptic island 8

Supplementary Note 5. Core bending within Fe island by pinning at the surface 10

Supplementary Note 6. Core position error using dI/dV data at fixed position and varying $B_{\|}$ 12

Supplementary Note 7. Approximations for micromagnetic simulations 14

Supplementary Note 8. Approximations for core path simulation 17

Supplementary Note 9. Errors in core path simulation and deduced pinning potential 19

Supplementary Note 10. Ab-initio calculations 20

Supplementary References 24

References 24

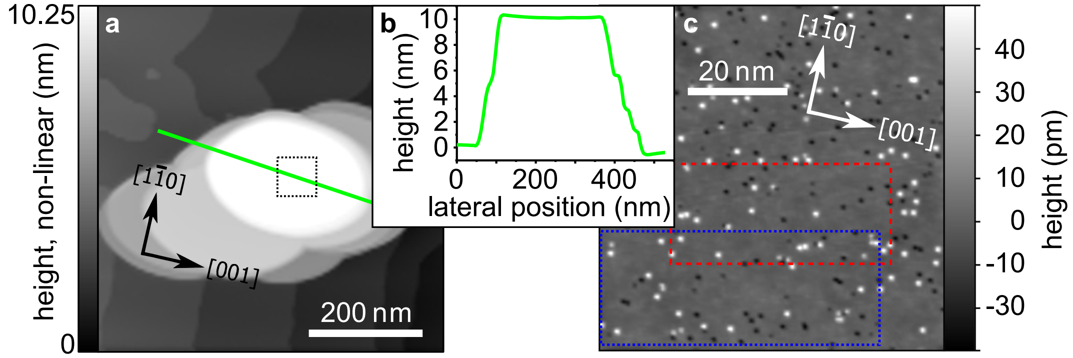

Supplementary Figure 1. **Investigated Fe island.** **a**, Topographic image of the island displayed in non linear gray-scale to enhance the visibility of substrate step edges. The island is imaged multiple times due to tip artifacts. **b**, Topographic profile along the green path in a. The average island height is 10 nm. **c**, Zoom into the area marked by the dashed box in a showing the adsorbates. Red box depicts the area imaged in Fig. 1e of the main text, blue box depicts the area imaged in Fig. 1f of the main text [1].

Supplementary Note 1: FE ISLAND

Supplementary Figure 1a shows an STM image of the Fe island that has been studied in Fig. 1b-f and Fig. 2 of the main text. Its size is $255 \times 165 \times 10\,\mathrm{nm}^3$. The crystallographic axes of the substrate as deduced from a low-energy electron diffraction pattern are added. The topographic image suffers from a multi-tip artifact that images the island several times. This does not influence spectroscopic measurements on the topmost imaged surface since the additional tips are a few nanometers away from that surface during its measurement.

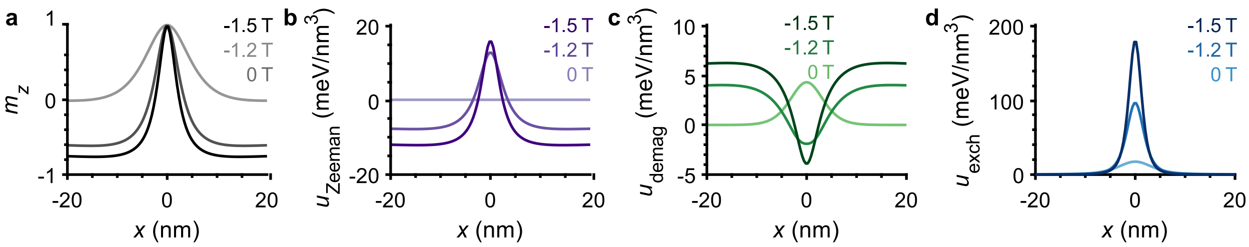

Supplementary Figure 2. **Vortex core energy densities.** **a**, Profiles of perpendicular magnetization $m_z = M_z/M_{\mathrm{sat}}$ of a simulated vortex core in a disk of height 10 nm and diameter 280 nm at $B_\perp$ according to legend. **b-d**, Profiles of Zeeman energy density, demagnetization energy density, and exchange energy density.

Supplementary Note 2: MICROMAGNETIC ENERGY DENSITIES OF VORTEX CORE

Magnetic vortex patterns are relaxed within the micromagnetic software package mumax[3] for a circular Fe island of thickness 10 nm and diameter 280 nm at perpendicular fields of $B_\perp = 0$ T, -1.2 T, and -1.5 T. The simulation space is discretized into $768 \times 768 \times 1$ cells of size $0.364 \times 0.364 \times 10\,\mathrm{nm}^3$. Magnetic parameters are set to saturation magnetization $M_{\mathrm{sat}} = 17$ kA/m, exchange stiffness $A_{\mathrm{ex}} = 21$ pJ/m, and zero magnetocrystalline anisotropy [2]. Spatially resolved energy densities of the Zeeman term, the demagnetization and the exchange are output by the software after relaxation of the magnetization pattern. Profiles through the vortex center of the cylindrical symmetric energy densities are shown together with profiles of the scaled out-of-plane magnetization m_z in Supplementary Fig. 2.

The m_z profiles (Supplementary Fig. 2a) largely map the experimentally observed dI/dV images, in particular, if the exact shape of the island is taken into account (section Supplementary Note 3). The exchange energy densities (Supplementary Fig. 2d) are much larger than the other two energy contributions. They, moreover, vary by approximately an order of magnitude with $B_\perp$, which results in a strong variation of pinning strength with $B_\perp$ for a defect with absent A_{ex}, as described in the main text.

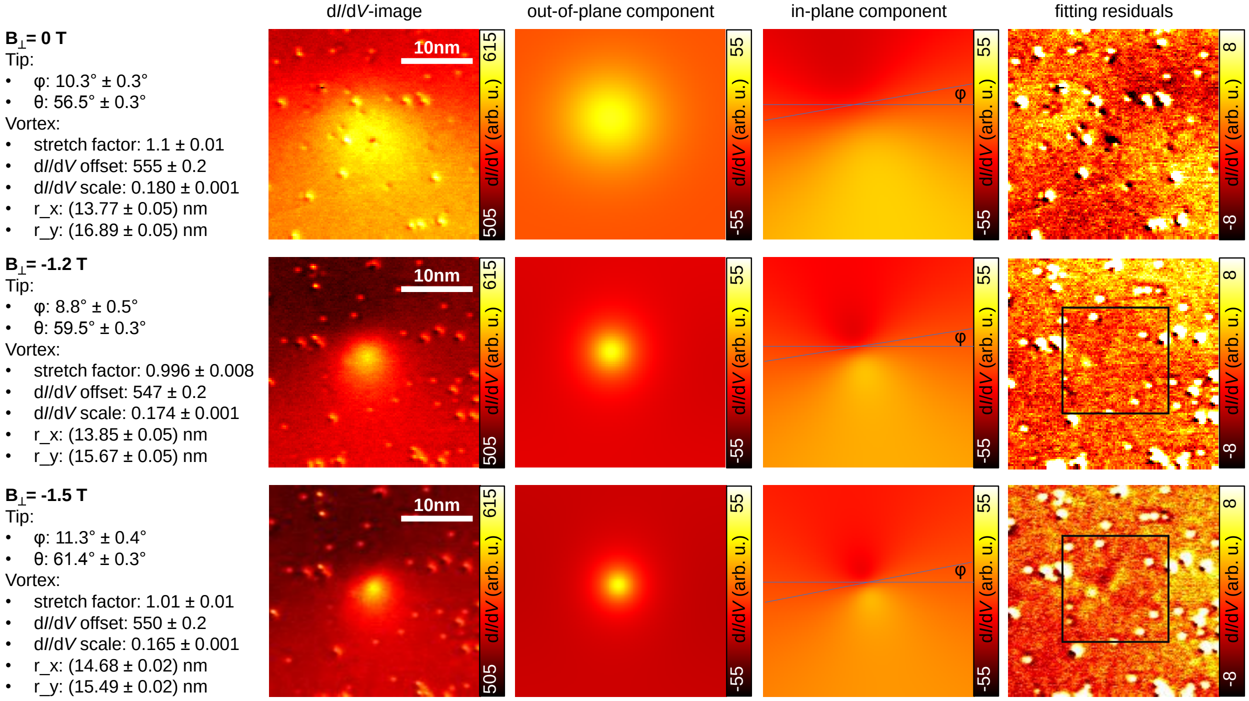

Supplementary Figure 3. **Fitting the dI/dV image of a vortex core by micromagnetically simulated m_z profiles.** Each row belongs to one $B_\perp$ as marked. The columns show (left to right): Fit parameters, original dI/dV images (same as Fig. 1b-d of main text), fitted out-of-plane magnetization component of dI/dV, fitted in-plane magnetization component of dI/dV, residual image. Note the larger contrast scale of the residual image by about an order of magnitude with respect to the other images. The fit parameters are azimuthal angle ϕ and polar angle θ of tip magnetization, a lateral scaling factor for the micromagnetically simulated images called stretch factor, a dI/dV offset and dI/dV scaling factor to account for non-spin-polarized dI/dV background and magnitude of spin polarized dI/dV signal, respectively [3], and the desired core center position (r_x, r_y) [1]. The black squares in the two lower right images mark the area where the fit is optimized. The fit angle ϕ is indicated in the images of the forth column.

Supplementary Note 3: CORE FITTING PROCEDURE

Supplementary Figure 3 visualizes the fitting procedure for dI/dV images of the vortex core as shown in Fig. 1b-d of the main text and again in the 2nd column of Supplementary Fig. 3.

The micromagnetic simulations employed for the core fits are conducted for an Fe island with thickness of 10 nm and lateral shape as determined by STM experimentally. The

island is discretized in cells of $0.359 \times 0.359 \times 1\,\mathrm{nm}^3$. The experimental dI/dV images and the micromagnetically simulated magnetization images are firstly interpolated to the same resolution. Moreover, defects are removed from the experimental image by a masking procedure prior to the fitting. Fit parameters are the two angles of the tip magnetization vector, the core position (r_x, r_y), a small lateral scale factor for the simulated images as well as the required offset and scale factor to transfer the dot product of magnetization vectors of tip and sample to the simulated dI/dV value [3].

The seven fit parameters are optimized towards minimum RMS deviation between simulated and measured $\mathrm{d}I/\mathrm{d}V$ map employing the MATLAB inbuilt trust-region-reflective least squares algorithm. At larger $B_\perp$, we only use the displayed black squares in the right column of Supplementary Fig. 3 for optimization such that we get more sensitive to the core region. The quality of the fits is visible in the most right column of Supplementary Fig. 3 showcasing the residual images that are obtained by subtracting the simulated dI/dV image from the experimental one. Only the adsorbates on the surface are visible with barely any magnetic contrast originating from the vortex core, even at the tenfold increased contrast scale of the residual images with respect to the experimental dI/dV images. The resulting fit parameters and confidence intervals are given in the left column of Supplementary Fig. 3.

The fit parameters firstly reveal a tip magnetization that slightly cants into the out-of plane direction with increasing $B_\perp$ as expected. Moreover, the stretch scale is very close to one at larger $B_\perp$, while deviating by 10% at $B_\perp = 0\,\mathrm{T}$. In line, the residual contrast surrounding the core is more pronounced at $B_\perp = 0\,\mathrm{T}$, where it features four areas of alternating bright and dark contrast. This is likely caused by the influence of adsorbates on the in-plane magnetization that prohibits a perfect fitting by the micromagnetic vortex simulated without defects. In line, the stretch factor at larger $B_\perp$ also deviates from one by $\sim 10\%$, if the adapting area is not reduced to the displayed square. The deduced dI/dV offset and dI/dV scale are very similar for all three $B_\perp$. The obtained large consistency of all fit parameters implies that the fits are reliable, in particular, at larger $B_\perp$, enabling a rather precise determination of the core center position of the vortex.

Via the extracted angle θ of tip magnetization, we, moreover, can discriminate the out-of-plane contrast and the in-plane contrast of the dI/dV images as displayed in the third and forth column of Supplementary Fig. 3 for the simulated dI/dV images. The in-plane angle of tip magnetization ϕ is additionally marked. The discrimination is used to display

an overlap of several vortex cores in one image as in Fig. 1e-f of the main text. To improve the visibility of each core, we subtract the in-plane contrast from the experimental dI/dV images. For Fig. 1f of the main text and the Supplementary Movie 2, we afterwards multiply the remaining out-of-plane contrast including defects by a Gaussian envelope centered at the vortex core center. This makes following the vortex core visually significantly more easy.

Supplementary Note 4: CORE MOVEMENT IN ELLIPTIC ISLAND

As described in the main text, the lateral core position $\mathbf{r}$ induced by $\mathbf{B}_{\|} = (B_x, B_y)$ follows $\mathbf{r}(\mathbf{B}_{\|}) = (\chi_{\text{free}} B_y, \chi_{\text{free}} B_x)$ with displacement rate χ_{free} for a circular magnetic island [4]. We assume a similar relation $\mathbf{r}(\mathbf{B}_{\|}) = (\chi_{x,\text{free}} B_y, \chi_{y,\text{free}} B_x)$ for the investigated elliptical island. This allows us to deduce target positions from $\mathbf{B}_{\|}$ by a shift $\mathbf{r} = (\chi_{x,\text{free}} B_y, \chi_{y,\text{free}} B_x)$ from the starting point $\mathbf{r}(\mathbf{0}\,\text{T}) = \mathbf{0}\,\text{nm}$. The validity of this assumption is verified by micromagnetic simulations revealing that a change of $\mathbf{B}_{\|}$ by $\Delta\mathbf{B}_{\|}$ results in nearly identical core shifts $\Delta\mathbf{r}$ independent of $\mathbf{B}_{\|}$. We employ a 3×3 grid of simulations with equidistant $\mathbf{B}_{\|}$ using the experimental island shape with cell size $0.36 \times 0.36 \times 10\,\text{nm}^3$ and magnetic parameters as displayed in Fig. 3a of the main text.

Supplementary Figure 4a−c show an m_z overlay of the resulting nine vortex cores with centers connected by colored vectors for each of the three experimental $B_{\perp}$. The bottom left vortex core is used as reference point with two lattice vectors (blue) to its nearest neighbors. These vectors set the displacement rates $\chi_{x,\text{free}}$ and $\chi_{y,\text{free}}$. Assuming constant $\chi_{i,\text{free}}$, the red vectors mark the lattice continuation that roughly hits the other calculated vortex cores. Zooming into the area of the upper right core (Supplementary Fig. 4d−f) reveals a remaining mismatch of $\sim 1\,\text{nm}$. This corresponds to a displacement error of $\sim 3\,\%$ on the full range of 37 nm of core movement, directly translating to an error of the anticipated constant χ_{free} in Fig. 2f−h of the main text. Note that the distance of the simulated core movement in Supplementary Fig. 4 is identical to the experimental one in Fig. 2f−h of the main text.

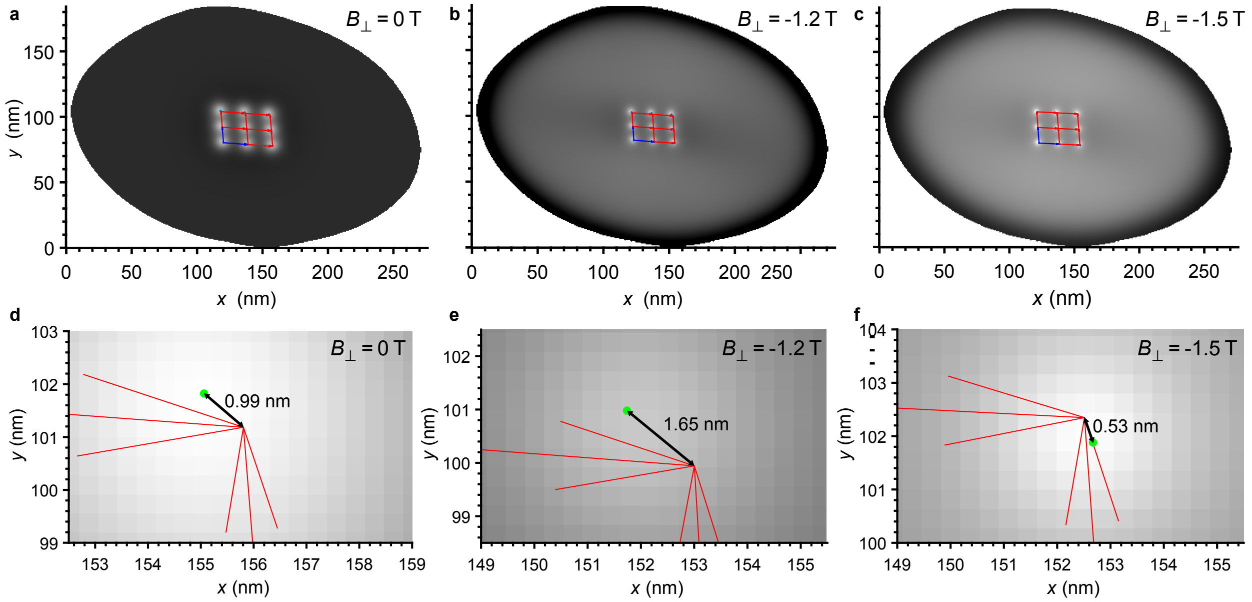

Supplementary Figure 4. **Validity of constant displacement rate by $B_{\|}$ in an elliptical island.** The program mumax^3 is used to simulate the movement of the vortex core within an island featuring the shape of the experimental one. Nine positions are targeted using a 3×3 grid of equidistant (B_x,B_y). **a-c**, Grayscale plots of cumulative m_z of all nine simulations featuring all nine vortex cores for each $B_\perp$. The contrast is adapted to minimum and maximum of m_z in each image individually. Blue vectors interconnect the lower left vortex core center to its two neighbors. Red vectors result from shifting the blue vectors in order to continue the lattice. **a**, $B_\perp = 0\,$T, B_x = 12/0/-12 mT, B_y = -12/0/12 mT. **b**, $B_\perp = -1.2\,$T, B_x = 8/0/-8 mT, B_y = -8/0/8 mT. **c**, $B_\perp = -1.5\,$T, B_x = 7/0/-7 mT, B_y = -7/0/7 mT. **d–f**, Zoom into the upper right vortex core area of the 3×3 grid in a–c, respectively. The green dots mark the simulated vortex core center deduced from the m_z maximum as found by spline interpolation. Red arrows are the end points of the continuation vectors from a–c. The mismatch between the vector addition (red arrows) and the simulated core positions is marked being 1–4 % of the full distance of movement of 37 nm.

Supplementary Note 5: CORE BENDING WITHIN FE ISLAND BY PINNING AT THE SURFACE

Spin polarized STM probes the magnetization of the surface layer that could be distinct from the magnetization in deeper layers. In particular, if the pinning center is at the surface only, the vortex core might bend towards its target position in deeper layers. Here, we show that the resulting vortex core bending is small.

We analyze micromagnetic simulations with vortex cores shifted from the island center. The shift is achieved by fixing m_z within 4×4 surface cells offset from the island center. The fixed m_z values are set to the values that are found in the core center for simulations without defects. The resulting cross section of m_z through the island (Supplementary Fig. 5a) is analyzed. We use cross sections slightly offset from the island center to avoid the cells with artificially fixed m_z. Supplementary Figure 5b shows deduced core positions (m_z maxima) evaluated for each layer separately. The core at $B_\perp = 0\,\mathrm{T}$ (-1.2 T, -1.5 T) is bent by 30 % (6.3 %, 2.7 %) of the average displacement from the island center. The bending at $B_\perp \neq 0\,\mathrm{T}$, where we observe pinning in the experiment, is well below 10% and, hence, barely changes the pinning energy. Such core bending is anyway included in our micromagnetic simulations of u_{exch} (Fig. 3g of the main text) and in the calculation of the parabolic potential E_{flex} for moving the vortex core away from its target (Supplementary Fig. 7a, Supplementary Fig. 8a).

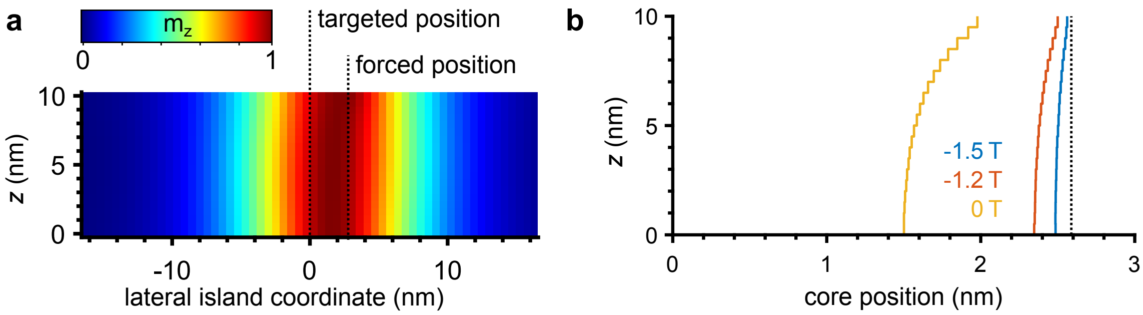

Supplementary Figure 5. **Core bending in Fe island.** **a**, Cross-sectional view of perpendicular magnetization m_z at $B_\perp = 0\,\mathrm{T}$ recorded for a plane that is 1 nm offset from the vortex core center. The circular island has thickness of 10 nm and diameter of 280 nm. The core is forced away from the island center by fixing m_z in 4×4 surface cells at 2.6 nm. **b**, Core position (m_z maximum) in each of the 20 layers of the simulated island for $B_\perp$ as labeled. The dotted black line depicts the position of frozen m_z.

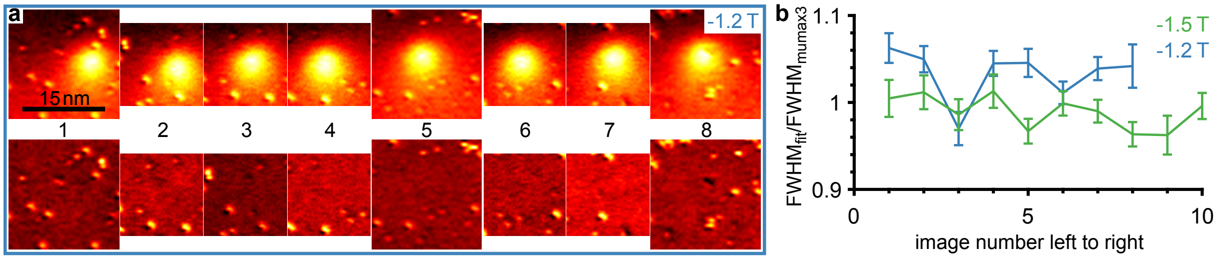

Supplementary Figure 6. **Core diameters for various core positions.** **a**, Top row: dI/dV images at $B_\perp = -1.2\,\mathrm{T}$ used to determine the 8 core positions marked in Fig. 2b of the main text. The images are fitted as described in section S3, resulting in the residuals as shown in the bottom row. The contrast of each image is scaled differently to optimize visibility. **b**, FWHM of the m_z distribution deduced via fit of the images of a (blue) and of 10 images recorded at $B_\perp = -1.5\,\mathrm{T}$ with core positions as marked in Fig. 2b of the main text (green). The FWHM obtained from the fit is scaled to the FWHM of a simulation without defects (called stretch factor in Supplementary Fig. 3) to ease comparison of the data at different $B_\perp$. The error bars correspond to 95 % confidence interval.

Supplementary Note 6: CORE POSITION ERROR USING dI/dV DATA AT FIXED POSITION AND VARYING $B_\parallel$

In Fig. 2 of the main text, we deduced the vortex core position from measuring dI/dV at fixed tip position, while varying $\mathbf{B}_\parallel$. This assumes a rigid vortex motion along a straight path. The assumption implies errors, since the vortex core shape could change by interactions with defects and the core is displaced from the straight path due to defect pinning as visible in Fig. 2b of the main text. These errors are discussed in the following.

To quantify the change of core shape, we analyze the core images along the core path of Fig. 2b of the main text (Supplementary Fig. 5a). The FWHM of m_z distribution is deduced from core fitting as described in section S3 . It is displayed in Supplementary Fig. 5b varying by about $\pm 5\,\%$ without any obvious trend within the error bars from the fitting procedure. Hence, core shape modifications during pinning are below 5 %. This value is regarded as error for the link between measured dI/d$V(B_\parallel)$ and core displacement (Fig. 2 of main text).

Moreover, the core path is deflected from the straight path by the defects. It exhibits RMS deviations perpendicular to the target path up to 1.3 nm (Fig. 2b of main text). This implies

two systematic errors. First, the path gets longer by the zigzag motion such that χ_{pinned} is underestimated by assuming a straight path. This error is estimated straightforwardly by using the measured path of Fig. 2b of the main text. The real path is by 5 % (0.3 %) longer than the straight path at $B_\perp = -1.5$ T (-1.2 T). For the estimate, we measure the largest angle between target path and direct lines between adjacent core positions to be $\sim 30°(\sim 7°)$ at $B_\perp = -1.5$ T (-1.2 T) (Fig. 2b, main text) and assume a normal distribution of such angles between adjacent core positions up to the maximum angle.

Second, perpendicular motion changes the sensed core magnetization at fixed tip position since the tip probes another part of the vortex. This error largely disappears for multiple pinning sites, since it either enhances or decreases χ_{pinned} by corresponding changes of dI/dV depending on the individual core center position with respect to the tip and the target path.

Supplementary Note 7: APPROXIMATIONS FOR MICROMAGNETIC SIMULATIONS

For simulated sweeps of $\mathbf{B}_{\parallel}$, as employed for Fig. 2f-h, Fig. 3e, and Fig. 4b of the main text, two approximations are used to reduce computational time. They are validated in the following.

As first approximation, instead of sweeping $\mathbf{B}_{\parallel}$, we shift the defect by $-\chi_{\mathrm{free}}(B_{\perp}) \cdot \mathbf{B}_{\parallel}$ through the vortex core. This requires that $E_{\mathrm{flex}}(\mathbf{r}_{\mathrm{vortex}} - \mathbf{r}_{\mathrm{target}})$, the displacement energy of the vortex around a target position $\mathbf{r}_{\mathrm{target}} = (x_{\mathrm{t}}, y_{\mathrm{t}})$, does not depend on $\mathbf{r}_{\mathrm{target}}$.

To show this, we simulate E_{flex} for $\mathbf{r}_{\mathrm{target}}$ either located in the center of the island or offset from it (main text, methods). We employ a grid with one cell in vertical direction for the sake of simplicity such that the core displacement is accomplished by a single cell of fixed $m_z = 1$ located away from $\mathbf{r}_{\mathrm{target}}$. It turned out that E_{flex} remained parabolic at all relevant distances of $\mathbf{r}_{\mathrm{target}}$ up to 30 nm from the center of the island. Supplementary Figure 7a displays the micromagnetically calculated $E_{\mathrm{flex}}(\mathbf{r}_{\mathrm{vortex}} - \mathbf{r}_{\mathrm{target}})$ for different $\mathbf{r}_{\mathrm{target}}$ along the target path in comparison with parabolic fits showcasing the nice agreement. The curvature of the parabola changed by 0.01 % (10 %) for distances of 5 nm (30 nm) from the island center. We conclude that the displacement of the vortex mostly depends on the relative distance to the defect, but only marginally on the absolute position of the core within the island. Hence, moving the defect instead of the vortex core is a reasonable approximation to deduce χ_{pinned} (Fig. 2f-h and Fig. 3e of the main text). Note that Fig. 2f-h of the main text cover only ± 8 nm such that the curvature error is well below 1%.

This agreement also justifies the assumption of a paraboloid for $E_{\mathrm{flex}}(\mathbf{r}_{\mathrm{vortex}} - \mathbf{r}_{\mathrm{target}})$ for the simulation of core movement in the disorder potential as shown in Fig. 4b of the main text. Deviations from the paraboloid in the direction perpendicular to the target path are even smaller, since the effective magnetization around the vortex is even less changed.

The independence of $E_{\mathrm{flex}}(\mathbf{r}_{\mathrm{vortex}} - \mathbf{r}_{\mathrm{target}})$ from $\mathbf{r}_{\mathrm{target}}$ is corroborated by a simplified analytic model assuming a rigid movement of vortex magnetization by $\mathbf{B}_{\parallel}$ [5]. This employs the magnetic displacement model for a magnetic cylinder discussed in the main text with potential energy $E(\mathbf{r}, \mathbf{B}_{\parallel}) = \frac{1}{2}k(x^2 + y^2) - k\chi_{\mathrm{free}}(B_y x + B_x y)$. The equation can be rewritten as $E(\mathbf{r}) = \frac{1}{2}k((x - x_{\mathrm{t}})^2 + (y - y_{\mathrm{t}})^2) + \frac{1}{2}k(x_{\mathrm{t}}^2 + y_{\mathrm{t}})^2$ with $x_{\mathrm{t}} = \chi_{\mathrm{free}} \cdot B_y$ and $y_{\mathrm{t}} = \chi_{\mathrm{free}} \cdot B_x$. Hence, moving $\mathbf{r}_{\mathrm{target}}$ on a circular island leads only to an offset in potential energy (second

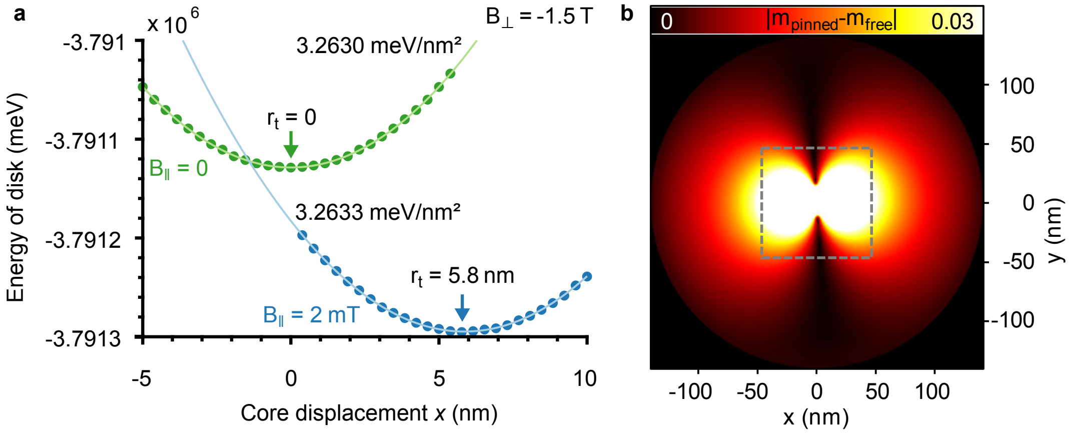

Supplementary Figure 7. **Validation of approximations in micromagnetic simulations. a**, Micromagnetically calculated potential energy of vortex core displacement for $B_\perp = -1.5\,\mathrm{T}$ and $B_y = 0\,\mathrm{T}$ (green dots) as well as $B_y = 2\,\mathrm{mT}$ (blue dots). The curvatures of the parabolic fits (solid lines) and the equilibrium positions r_t are marked. The micromagnetic simulations consider a cylindrical island of 280 nm diameter and 10 nm height discretized in cells of $0.38 \times 0.38 \times 10\,\mathrm{nm}^3$. Displacements are realized by fixing one cell to $m_z = 1$ away from the target position $\mathbf{r}_{\mathrm{target}}$. **b**, Absolute difference of m_z between an unpinned vortex core and a core pinned at $\mathbf{r}_{\mathrm{vortex}} = (3.6, 0)\,\mathrm{nm}$ away from the island center ($B_\perp = -1.5\,\mathrm{T}$). Same island size and cell size as in a. Only the area in the dashed box is used for full simulations of vortex-defect-interactions, while the remaining area is approximated by a demagnetization field independent of core position. The normalized magnetization difference within the dashed grey box reaches up to 1.8, while it is below 3 % outside of the box [1].

term), but does not affect the potential curvature k or the potential shape.

As second approximation, we crop the simulation area to $256 \times 256 \times 20$ cells and add the demagnetization field of the missing exterior by hand leading to an effective magnetic field $\mathbf{B}_{\mathrm{eff}}(\mathbf{r}) = \mathbf{B}_\perp + \mathbf{B}_{\mathrm{demag,exterior}}(\mathbf{r})$. $\mathbf{B}_{\mathrm{demag,exterior}}(\mathbf{r})$ is calculated once for an unperturbed vortex without defects at $\mathbf{B}_{||} = \mathbf{0}\,\mathrm{T}$ and is fixed afterwards for all other simulations. This is possible, since we always use $\mathbf{B}_{||} = \mathbf{0}\,\mathrm{T}$ and, thus, $\mathbf{r}_{\mathrm{target}} = \mathbf{0}\,\mathrm{nm}$ via the first approximation. The small core displacement resulting from pinning forces by defects changes the magnetization only within the cropped area significantly. Supplementary Figure 7b shows the spatially resolved absolute difference in magnetization between a vortex core located at $\mathbf{r}_{\mathrm{vortex}} = \mathbf{r}_{\mathrm{target}} = \mathbf{0}\,\mathrm{nm}$ and a core moved by pinning to $\mathbf{r}_{\mathrm{vortex}} = (3.6, 0)\,\mathrm{nm}$ at $B_\perp = -1.5\,\mathrm{T}$. This

displacement is larger than any displacement observed experimentally due to defects. The scaled magnetization m_z outside the fully simulated area (gray box) varies by less than 3 % strongly decaying away from the square. As shown in section Supplementary Note 2, the general influence of demagnetization on the vortex core energy is small. Hence, the resulting error of using an unmodified $\mathbf{B}_{\mathrm{demag,exterior}}(\mathbf{r})$ is likely negligible.

Supplementary Note 8: APPROXIMATIONS FOR CORE PATH SIMULATION

To emulate the vortex core path at varying $\mathbf{B}_{\parallel}$, we determine its lateral position by potential energy minimization within a potential landscape given by defects as described in the main text. The potential energy firstly consists of the potential $E_{\text{flex}}(\mathbf{r}_{\text{core}} - \mathbf{r}_{\text{target}})$ describing the energy cost to move the core away from its target position $\mathbf{r}_{\text{target}}(\mathbf{B}_{\parallel})$ in the absence of defects. Secondly, the pinning potentials centered at each adsorbate $E_{\text{i,pin}}(\mathbf{r}_{\text{vortex}} - \mathbf{r}_{\text{i,adsorbate}})$ contribute to the potential energy. For both potential parts, we use approximations that enable easier computation.

E_{flex} is deduced from forcing the vortex core away from $\mathbf{r}_{\text{target}}$. Therefore, m_z is fixed within $4 \times 4 \times 1$ simulation cells at the surface positioned away from $\mathbf{r}_{\text{target}}$ to the m_z values of a defect-free vortex core center. Subsequently, the vortex energy at the resulting core position is calculated. This mimics forcing the core away from $\mathbf{r}_{\text{target}}$ by a defect. Such movement differs from movements via $\mathbf{B}_{\parallel}$ regarding the change of magnetization in the surrounding of the core. The required unphysical area of fixed magnetization barely changes the vortex energy. To estimate the corresponding error, we employed a second relaxation step while fixing the magnetization obtained from the first relaxation in all cells except of the priorily fixed ones and one additional ring of cells surrounding them. For the largest core displacement observed at $B_{\perp} = -1.5\,\text{T}$, the potential energy changes by only $1.8\,\%$ due to this second relaxation step. Hence, the energy error of fixing m_z in a few cells is well below $2\,\%$. Afterwards, the resulting $E_{\text{flex}}(\mathbf{r}_{\text{core}} - \mathbf{r}_{\text{target}})$ is fitted by a parabola (Supplementary Fig. 8a). The fit exhibits a negligible RMS deviation of $0.03\,\text{meV}$ to the micromagnetic data for the largest displacements observed experimentally. Thus, we used a parabola for $E_{\text{flex}}(\mathbf{r}_{\text{core}} - \mathbf{r}_{\text{target}})$ further on.

For the pinning potentials $E_{\text{i,pin}}(\mathbf{r}_{\text{vortex}} - \mathbf{r}_{\text{i,adsorbate}})$, identical for each i, we superposed a repelling Gaussian and the scaled exchange energy density $u_{\text{exch}}(\mathbf{r})$ of the core as described in the main text. To increase computational speed, we employ an analytic representation of $u_{\text{exch}}(\mathbf{r})$, based on an analytic approximation of $m_z(\mathbf{r})$:

$$m_z(\mathbf{r}) = a + (1 - a)/\cosh(2 \cdot \text{arcosh}(2) \cdot r/\text{FWHM}) \tag{1}$$

with a being the magnitude of m_z in the surrounding of the vortex core and the width of

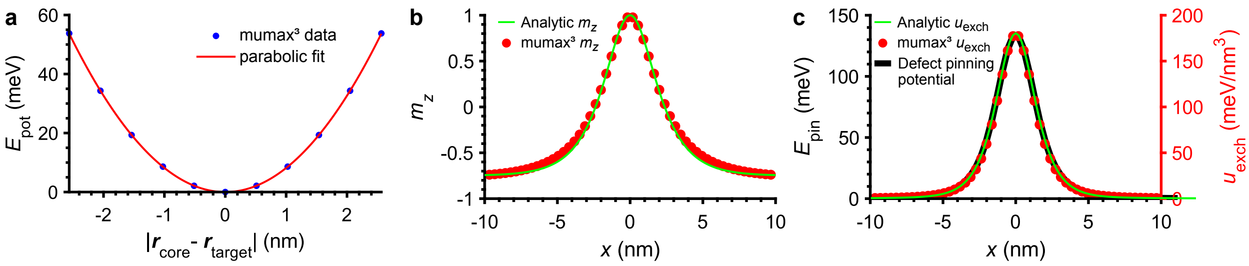

Supplementary Figure 8. **Approximations for core path simulation.** **a**, Potential energy of vortex core without defects as a function of distance between core position $\mathbf{r}_{\text{core}}$ and target position $\mathbf{r}_{\text{target}}$, $B_\perp = -1.5\,\text{T}$. The simulation is based on a circular Fe islands (thickness: 10 nm, diameter: 280 nm) discretized into cells of size $0.364 \times 0.364 \times 0.5\,\text{nm}^3$. For each data point, the core is forced away from $\mathbf{r}_{\text{target}} = (0,0)$ by fixing m_z in $4 \times 4 \times 1$ cells at the surface. The parabolic fit (red line) yields negligible deviations from the data point of $0.03\,\text{meV}_{\text{RMS}}$. **b**, m_z profile of vortex core according to micromagnetic simulation by mumax^3 at $B_\perp = -1.5\,\text{T}$ and to the analytic description of eq. (1) with FWHM$= 4.2\,\text{nm}$ and $a = -0.8$. **c**, Vortex core exchange energy density u_{exch} from the analytic description (eq. 2) with same parameters as in a and from mumax^3. The black line depicts the inverted pinning potential for a defect with absent A_{ex} (Fig. 3g of main text), $B_\perp = -1.5\,\text{T}$.

the core FWHM. This leads to

$$\begin{aligned} u_{\text{exch}}(\mathbf{r}) &= A_{\text{ex}} \cdot \Big(\quad \nabla \mathbf{m}(\mathbf{r}) \Big)^2 \\ &= A_{\text{ex}} \cdot \Big(\quad \big(-b \cdot \tanh(r/c) \cdot \text{sech}(r/c)/c \big)^2 \\ &\qquad + \big(1 - (b \cdot \text{sech}(r/c) + a)^2 \big) / r^2 \\ &\qquad + \big(b \cdot \tanh(r/c) \cdot \text{sech}(r/c) \cdot (b \cdot \text{sech}(r/c) + a)/c/\sqrt{1 - (b \cdot \text{sech}(r/c) + a)^2} \big)^2 \Big) \end{aligned} \tag{2}$$

with $b = 1 - a$ and $c = \text{FWHM}/(2 \cdot \text{acosh}(2))$.

Supplementary Figure 8c compares $u_{\text{exch}}(\mathbf{r})$ from mumax^3 with the analytic description as best fit by adapting a and FWHM. Excellent agreement is achieved with rms deviation of $0.6\,\text{meV/nm}^3$ only. The comparison of m_z profiles is shown in Supplementary Fig. 8b. The reversed pinning potential for a defect with suppressed A_{ex} within $1.1 \times 1.1 \times 0.5\,\text{nm}^3$ (Fig. 3g of main text) is added to Supplementary Fig. 8c. Obviously, the relatively small defect simply tracks $u_{\text{exch}}(\mathbf{r})$ such that the scaled analytic $u_{\text{exch}}(\mathbf{r})$ can be used to mimic the attractive part of the defect potential for the core path simulation.

Supplementary Note 9: ERRORS IN CORE PATH SIMULATION AND DEDUCED PINNING POTENTIAL

The most severe error in core path simulation results from the remaining uncertainty in the adaption of the core shape at a defect. As shown in Supplementary Fig. 6b, the FWHM of the m_z profile fluctuates by $\pm 5\%$. This translates via eqs. (1) and (2) (section Supplementary Note 8) to an error of $\pm 5\%$ in the FWHM of u_{exch}, hence, influencing the pinning potential analogously by construction. The other energy errors are significantly smaller, namely, the error due to determination of E_{flex} via fixing m_z in $4 \times 4 \times 1$ simulation cells ($\leq 1.8\%$, section Supplementary Note 8), the error due to determination of E_{flex} by moving the defect instead of the vortex core ($< 1\,\%$, section Supplementary Note 7), the error due to the parabolic fit of E_{flex} ($< 0.1\%$, section Supplementary Note 8) and the error due to the cropping procedure (likely negligible, section Supplementary Note 7).

Another source of error is more difficult to quantify. It is given by uncertainties in the determined core positions that are non-linearly linked to the deduced defect potential. This includes the missing knowledge on the true target path due to the fact that start and end point of the path of the vortex core are influenced by defects, too. The adaption of these points in our fitting routine reveals deviations by $1-2\,\mathrm{nm}$ on the full length of $40\,\mathrm{nm}$ in line with typical excursion lengths from the straight path due to defects. A similar deviation results from the anticipated straight target path in an elliptic island being incorrect by $1-2\,\mathrm{nm}$ on the path of $40\,\mathrm{nm}$, too (section Supplementary Note 4). Other position errors are much smaller such as uncertainties in core center positions deduced from the fitting of noisy images ($< 0.1\,\mathrm{nm}$, section Supplementary Note 3), uncertainties in the overlap of adjacent images of the vortex core ($< 0.1\,\mathrm{nm}$) and creep and drift effects within the images ($\sim 0.1\,\mathrm{nm}$, [6]).

Importantly, the main errors can be improved, in principle, via reducing the defect density, such that the distance between defects is significantly larger than the core diameter. Then, the influence of a single defect on the core shape can be probed in detail and start and end points of the target path can be chosen far away from any defect. Subleading errors can be reduced by more elaborate micromagnetic simulations.

Supplementary Note 10: AB-INITIO CALCULATIONS

We performed ab-initio based calculations of Cr- and O-adatoms deposited on an Fe(110) surface using density functional theory (DFT) as implemented in the full-potential Korringa-Kohn-Rostoker Green function (KKR-GF) method [7, 8]. Relativistic effects are taken into account via the scalar relativistic approach with the self-consistent inclusion of the spin orbit coupling as a perturbation. The exchange correlation potential is treated in the local spin density approximation as parametrized by Vosko, Wilk and Nusair [9]. Instead of seeking for the wave function of the system, the KKR-GF method aims primarily at calculating the Green function using multiple scattering theory by solving the Dyson equation:

$$\mathcal{G} = \mathcal{G}_0 + \mathcal{G}_0 \Delta V \mathcal{G}. \tag{3}$$

This enables, e.g., to describe impurities deposited on a pristine substrate using an embedding scheme. Indeed, the previous Dyson equation can be solved in real space by obtaining the Green function $\mathcal{G}$ of the investigated material by knowing the Green function $\mathcal{G}_0$ of the perfect Fe(110) substrate and ΔV, the potential change induced by the adatom. Once the Green function is obtained, the electronic and magnetic properties are deduced by extracting, e.g., charge and spin densities, local density of states, and magnetic exchange interactions.

The Fe(110) substrate with a lattice constant of $a_{\text{lat}} = 286\,\text{pm}$ is simulated considering a slab containing 12 layers of Fe with enough vacuum layers surrounding it, six on each side of the slab. After relaxing the atomic positions at the surface, leading to values in accordance with [10], we solve the previous Dyson equation for a real-space impurity cluster. This cluster has a diameter of 6 lattice constants and consists of the adsorbate and 150 Fe atoms from the substrate (Supplementary Fig. 9a/c). The adsorbates, O or Cr, are located in the long bridge position at a distance of 103 pm above the surface as known for O [10–13] and assumed to be identical for Cr.

Without the adsorbate, the average magnetic moment of the Fe atoms is 2.65 μ_{B}. With O (Cr), the closest Fe moment decreases to 1.68 μ_{B} (0.68 μ_{B}) while the substrate without considering the adsorbate experiences a cumulative reduction of the magnetization by 1.7 μ_{B} (4.6 μ_{B}).

The change of the anisotropy due to the oxygen adsorbate was calculated by the energy difference $\Delta E_{\alpha-\beta} = (E_\alpha^{\text{with O}} - E_\beta^{\text{with O}}) - (E_\alpha^{\text{without O}} - E_\beta^{\text{without O}})$, where α and β denote the

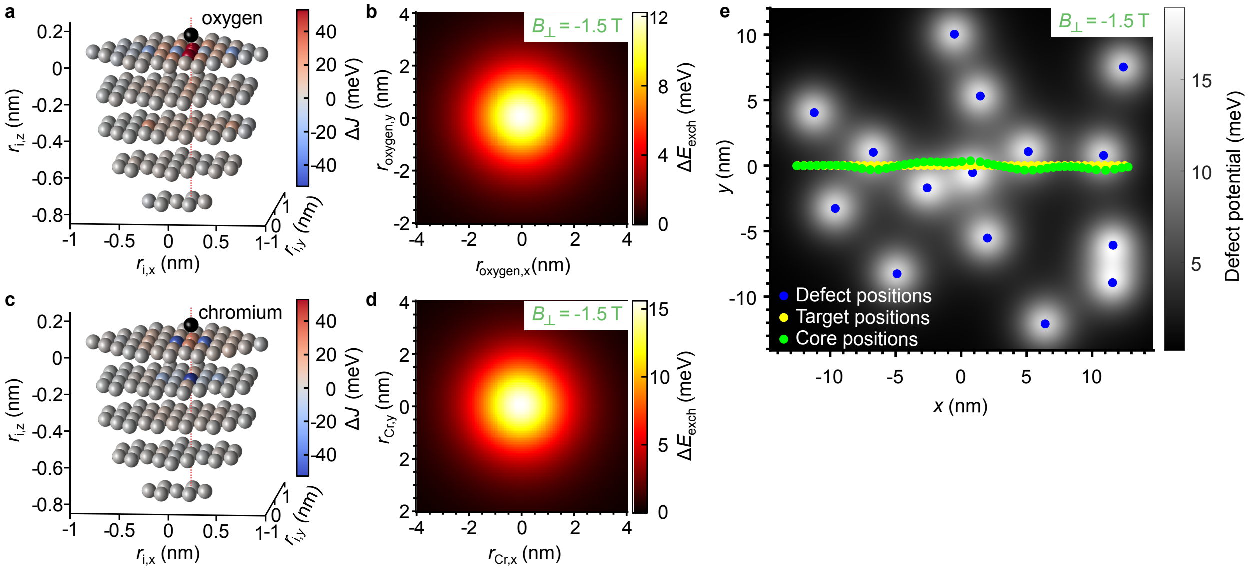

Supplementary Figure 9. **Ab-initio based vortex core energy around O and Cr adsorbates.** **a**, Change of the site dependent $J_i = \sum_j J_{ij}/2$ for an Fe-cluster due to adding an O adsorbate (black) at a long bridge position. The difference $\Delta J_i = J_i^{\text{with O}} - J_i^{\text{without O}}$ is color coded on the grey spheres representing the Fe atoms, i.e., red (blue) color indicates a stronger (weaker) ferromagnetic coupling of the atom at r_i to the other Fe atoms. **b**, Resulting exchange energy potential of the vortex for varying vortex core position with respect to the O position, $B_\perp = -1.5\,\text{T}$. The potential is set to zero far away from the O atom. For each pixel of the potential, the magnetic moments $\mathbf{m}_i(\mathbf{r}_i)$ of a micromagnetically obtained vortex without defect are used to calculate $\Delta E_{\text{exch}} = \sum_{i<j}(J_{ij}^{\text{with O}} - J_{ij}^{\text{without O}})(\mathbf{m}_i \cdot \mathbf{m}_j)$ for the respective vortex core center position with respect to the O position. **c**, Analogous to a, but with Cr adsorbate. **d**, Analogous to b, but with Cr adsorbate. Exchange coupling between Cr and the substrate atoms is taken into account. **e**, Simulated vortex core path (green) at $B_\perp = -1.5\,\text{T}$ employing 15 Cr defects that are randomly placed within $10 \times 10\,\text{nm}^2$ according to the defect density of the experiment. The resulting disorder potential is displayed as grey scale as deduced from superposing the defect potential of d for each adsorbate. The target path (yellow) consists of 50 equidistant positions along $y = 0$.

orientation of a ferromagnetic spin configuration along [001] (x-axis), [1$\bar{1}$0] (y-axis) or [110] (z-axis). Hence, $\Delta E_{\alpha-\beta} > 0$ indicates that the easy axis turns towards the β-direction when putting the O-adatom on top. The calculated values are $\Delta E_{x-z} = -0.68\,\text{meV}$, $\Delta E_{y-z} = -0.82\,\text{meV}$ and $\Delta E_{x-y} = 0.13\,\text{meV}$, i.e., an in-plane magnetization along y is favoured by the

O-adatom. Importantly, these energies are much lower than the experimentally observed pinning energies ($\sim$ 100 meV) discarding any influence of the anisotropy energy on the pinning.

Utilizing a mapping procedure based on infinitesimal rotation of the magnetic moments [14, 15], the magnetic exchange interactions J_{ij} of an isotropic Heisenberg Hamiltonian $\mathcal{H}_{\text{exc}} = -\sum_{i<j} J_{ij}\, \mathbf{m}_i \cdot \mathbf{m}_j$, are extracted from the ab-initio calculations, where $\mathbf{m}_i$ and $\mathbf{m}_j$ are the unit vectors of the magnetic moments of the ith and j^{th} atom, respectively.

A comparison of exchange parameters with and without adsorbate reveals that the cumulative exchange interaction is enhanced around both types of adatoms. Thus, we observe a global exchange stiffening. Supplementary Figure 9a shows a 3D map of the difference of the site dependent exchange parameter $\Delta J_i = J_i^{\text{with O}} - J_i^{\text{without O}}$ where $J_i = \sum_j J_{ij}/2$. The Fe atoms nearest to the adsorbate along [1-10] (y-axis) exhibit a stiffening of the exchange interaction, while the exchange interaction along [001] (x-axis) gets weaker, but by a smaller amount. The same is shown in Supplementary Fig. 9c for the Cr adsorbate, where stiffening along [1-10] is weaker and weakening along [001] is more pronounced than for the O adsorbate. The accumulated change in exchange energy amounts to $\Delta E_{\text{exch}} = 217$ meV (86 meV) for O (Cr) including the contribution of the Cr adatom of -34 meV. Since the exchange energy is increased in total (stiffening), a non-collinear magnetic texture as in the vortex core gains energy, if located away from the adsorbate, eventually leading to vortex core repulsion.

To reveal the interaction profile between adsorbates and vortex core, we employ J_{ij} as obtained from DFT and calculate the exchange energy via $\mathcal{H}_{\text{exc}}$ with the directions of the magnetic moments $\mathbf{m}_i$ set by the micromagnetically simulated vortex core profile. Changing the vortex core position with respect to the adsorbate reveals the interaction potentials as shown for $B_\perp = -1.5$ T in Supplementary Fig. 9b and d. The shape of the two potentials is identical with slightly different amplitude of 12 meV (15.5 meV) for the O (Cr) adatom. This amplitude is still an order of magnitude lower than in the experiment (Fig. 4c, main text).

Nevertheless, assuming the Cr induced interaction potential (Supplementary Fig. 9d), we simulated a vortex core path for randomly distributed Cr defects with density as in the experiment (Supplementary Fig 9e). The simulation procedure is identical to the one employed for Fig. 4b of the main text. The resulting core path (green) at $B_\perp = -1.5$ T

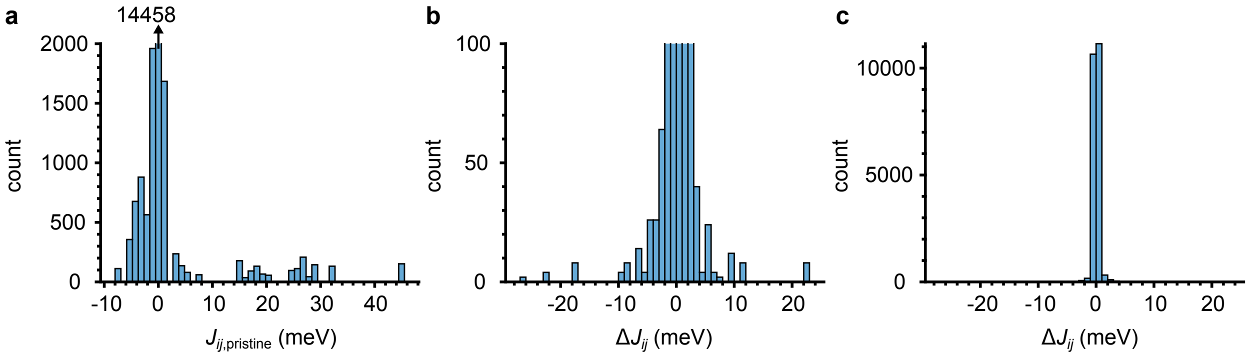

Supplementary Figure 10. **Histograms of exchange interactions between Fe atoms.** **a**, Histogram of all J_{ij} between the Fe atoms of the pristine substrate up to a distance of 6 lattice parameters around the site where O will be embedded. **b**, Histogram of $\Delta J_{ij} = J_{ij}^{\text{with O}} - J_{ij}^{\text{without O}}$, i.e., the changes of J_{ij} due to the O adsorbate for the same atoms as in a. **c**, Same as b but displayed at a different scale.

deviates by up to 600 pm from the straight target path (yellow). Such a deviation can be recorded by spin polarized STM and showcases that single adsorbates can alter the vortex path for a core size consisting of $\sim 10^4$ Fe atoms.

However, the much stronger excursions from the target path observed in the experiment can not be explained by this simulation. One origin of the discrepancy could be different values of J_{ij} than calculated via DFT. Supplementary Figure 10 shows histograms of the exchange parameter J_{ij} for the Fe atoms of the pristine substrate (Supplementary Fig. 10a) as well as of the change of the exchange parameters ΔJ_{ij} due to adding an O adsorbate (Supplementary Fig. 10b, c). The changes of J_{ij} are partly as large as J_{ij} itself. They, moreover, exhibit nearly as much reduction as increase of J_{ij}. In line, the accumulated $\sum \Delta J_{ij} = 217\,\text{meV}$ amounts to only 10% of the accumulated absolute energy change $\sum |\Delta J_{ij}| = 2.5\,\text{eV}$. This showcases that details in the interaction strengths J_{ij} including sign changes can modify the accumulated exchange energy significantly via subtraction of two similarly large numbers.

Other possible origins of the discrepancy are already mentioned in the main text. Firstly, the structural position of the adsorbate might not be correctly described in the DFT calculations again changing ΔJ_{ij} in detail. Secondly, the adsorbate might pinpoint to a particular strain field that might originate from the growth procedure and offers preferential adsorption sites.

SUPPLEMENTARY REFERENCES

---

[1] C. Holl, *High frequency STM and spin polarized STM on magnetic vortices*, Ph.D. thesis, RWTH Aachen University (2018).

[2] A. Wachowiak, J. Wiebe, M. Bode, O. Pietzsch, M. Morgenstern, and R. Wiesendanger, Science **298**, 577 (2002).

[3] M. Bode, Rep. Prog. Phys. **66**, 523 (2003).

[4] R. Badea and J. Berezovsky, Phys. Rev. Appl. **5**, 064003 (2016).

[5] M. Rahm, J. Stahl, W. Wegscheider, and D. Weiss, Appl. Phys. Lett. **85**, 1553 (2004).

[6] T. Mashoff, M. Pratzer, and M. Morgenstern, Rev. Sci. Instr. **80**, 053702 (2009).

[7] N. Papanikolaou, R. Zeller, and P. H. Dederichs, J. Phys.: Cond. Mat. **14**, 2799 (2002).

[8] D. Bauer, *Development of a relativistic full-potential first-principles multiple scattering Green function method applied to complex magnetic textures of nano structures at surfaces*, Ph.D. thesis, RWTH Aachen University (2014).

[9] S. H. Vosko, L. Wilk, and M. Nusair, Can. J. Phys. **58**, 1200 (1980).

[10] T. Ossowski and A. Kiejna, Surf. Sci. **637-638**, 35 (2015).

[11] T. Miyano, Y. Sakisaka, T. Komeda, and M. Onchi, Surf. Sci. **169**, 197 (1986).

[12] M. Getzlaff, J. Bansmann, and G. Schönhense, J. Magn. Magn. Mater. **192**, 458 (1999).

[13] M. Eder, K. Terakura, and J. Hafner, Phys. Rev. B **64** (2001), 10.1103/physrevb.64.115426.

[14] A. I. Liechtenstein, M. I. Katsnelson, and V. A. Gubanov, J. Phys. F: Met. Phys. **14**, L125 (1984).

[15] A. Liechtenstein, M. Katsnelson, V. Antropov, and V. Gubanov, J. Magn. Magn. Mater. **67**, 65 (1987).
