# Supplementary material for: Probing the pinning strength of magnetic vortex cores with sub-nanometer resolution
Source: Nat Commun. 2020 Jun 5;11:2833. doi: 10.1038/s41467-020-16701-y (PMC7275073; doi:10.1038/s41467-020-16701-y)
Supplement: Supplementary file 3 — Description of Additional Supplementary Files [file 41467_2020_16701_MOESM3_ESM.pdf]

Description of Additional Supplementary Files

Supplementary Movie 1

Supplementary Movie 1 sequentially shows 45 d*I*/dV-images recorded at $B_\perp = -1.5$ T, while moving the vortex core by 44 equidistant $\boldsymbol{B}_\parallel$ steps with $\Delta\boldsymbol{B}_\parallel =$ (136,-227) µT. These images are also used to determine the core positions shown in Fig. 1f of the main text. Each d*I*/d*V* image covers an area of 15x15 nm². Experimentally, 60x60 pixels are recorded at $V = -2$ V, $I = 1$ nA and modulation voltage of $50mV_{RMS}$. To optimize visibility, additional interpolated pixels are displayed in the movies. The scan frame center is moved linearly between adjacent images by a vector deduced from centering the core in initial and final image.

Supplementary Movie 2

Supplementary Movie 2 shows the same data as Supplementary Movie 1 in different color scale and overlaid on a separately measured topography of the whole area. Here, the d*I*/d*V*-images are displayed after subtracting the contrast originating from in-plane magnetization and multiplying the image with a Gaussian intensity profile as described in Supplementary Note 3. Additional minor shear and stretch transformations by ~1% are applied to remove the effects of piezo creep.
